# Metabolic Model of the *Phytophthora infestans*-Tomato Interaction Reveals Metabolic Switches during Host Colonization

**DOI:** 10.1128/mBio.00454-19

**Published:** 2019-07-09

**Authors:** Sander Y. A. Rodenburg, Michael F. Seidl, Howard S. Judelson, Andrea L. Vu, Francine Govers, Dick de Ridder

**Affiliations:** a Laboratory of Phytopathology, Wageningen University, Wageningen, the Netherlands; b Bioinformatics Group, Wageningen University, Wageningen, the Netherlands; c Department of Microbiology and Plant Pathology, University of California Riverside, Riverside, California, USA; Virginia Tech; Cornell University

**Keywords:** *Phytophthora infestans*, metabolic modeling, metabolism, oomycetes, tomato

## Abstract

The oomycete pathogen Phytophthora infestans causes potato and tomato late blight, a disease that is a serious threat to agriculture. P. infestans is a hemibiotrophic pathogen, and during infection, it scavenges nutrients from living host cells for its own proliferation. To date, the nutrient flux from host to pathogen during infection has hardly been studied, and the interlinked metabolisms of the pathogen and host remain poorly understood. Here, we reconstructed an integrated metabolic model of *P. infestans* and tomato (Solanum lycopersicum) by integrating two previously published models for both species. We used this integrated model to simulate metabolic fluxes from host to pathogen and explored the topology of the model to study the dependencies of the metabolism of *P. infestans* on that of tomato. This showed, for example, that *P. infestans,* a thiamine auxotroph, depends on certain metabolic reactions of the tomato thiamine biosynthesis. We also exploited dual-transcriptome data of a time course of a full late blight infection cycle on tomato leaves and integrated the expression of metabolic enzymes in the model. This revealed profound changes in pathogen-host metabolism during infection. As infection progresses, *P. infestans* performs less *de novo* synthesis of metabolites and scavenges more metabolites from tomato. This integrated metabolic model for the *P. infestans*-tomato interaction provides a framework to integrate data and generate hypotheses about *in planta* nutrition of *P. infestans* throughout its infection cycle.

## Abstract (precis)

Late blight disease caused by the oomycete pathogen *Phytophthora infestans* leads to extensive yield losses in tomato and potato cultivation worldwide. To effectively control this pathogen, a thorough understanding of the mechanisms shaping the interaction with its hosts is paramount. While considerable work has focused on exploring host defense mechanisms and identifying *P. infestans* proteins contributing to virulence and pathogenicity, the nutritional strategies of the pathogen are mostly unresolved. Genome-scale metabolic models (GEMs) can be used to simulate metabolic fluxes and help in unravelling the complex nature of metabolism. We integrated a GEM of tomato with a GEM of *P. infestans* to simulate the metabolic fluxes that occur during infection. This yields insights into the nutrients that *P. infestans* obtains during different phases of the infection cycle and helps in generating hypotheses about nutrition *in planta*.

## INTRODUCTION

Plants and pathogens maintain a complex relationship that generally involves the secretion of effector proteins by the pathogen to manipulate plant cell processes and the scavenging of nutrients from the host by the pathogen to support its growth and proliferation (1). While increasing knowledge has been gained on secreted effector proteins that facilitate host colonization (2), the nature of pathogen nutrition remains underexplored. A class of organisms comprising important plant and animal pathogens is the oomycetes (3). These share morphological characteristics with fungi, yet belong to the Stramenopiles, a eukaryotic lineage that besides oomycetes also includes diatoms and brown algae (4). The most well-known oomycete is Phytophthora infestans, the causal agent of late blight disease on tomato and potato, which leads to significant yield losses worldwide (5). *P. infestans* is challenging to control. The pathogen has a highly flexible genome facilitating rapid adaptation to control strategies, be it resistant cultivars or chemical agents (6), and its profuse sporulation causes P. infestans to spread extremely fast (7). Hence, there is a continuous quest for novel more durable control strategies.

*P. infestans* sporangia are aerially dispersed, and after landing on a plant surface, these can release flagellate zoospores (8). These encyst and germinate to form a germ tube with an appressorium to penetrate epidermal cells of the plant. From there, *P. infestans* colonizes the mesophyll; hyphae grow in the apoplast while forming intracellular feeding structures called haustoria. Both the apoplastic hyphae and haustoria provide close contact with the plant, facilitating the exchange of effectors and nutrients (9). Oomycetes are considered osmotrophs that extracellularly catabolize complex host polymers, such as proteins, sugars, and fatty acids, using an arsenal of secreted enzymes, followed by the import of degraded nutrients into the cell (10). Moreover, *P. infestans* is a hemibiotrophic pathogen that requires viable host cells during the initial stages of the infection cycle, the so-called biotrophic phase with minimal symptoms. Typically, after 3 to 6 days, this biotrophic phase is followed by a necrotrophic phase during which the lesion becomes necrotic and new sporangia emerge (11, 12). This implies that the physiology of the host tissue changes throughout the infection cycle, and consequently, also the nutrients available for the pathogen (13). Conceivably, *P. infestans* fine-tunes its metabolism to available nutrients, for example, by regulating the expression of enzyme-encoding genes through catabolite repression and/or substrate induction (14, 15).

The *P. infestans* metabolism is remarkably dynamic. Transcriptome-based studies revealed significant differences in the transcript abundances of enzyme-encoding genes throughout the asexual and sexual lifecycles and during plant infection (16–19). While these studies provide detailed insight into the potential dynamics of metabolic enzymes, the insight gained into the overall characteristics of the cell metabolism is very limited. Importantly, cell metabolism is not a static framework of reactions and pathways but adapts to different environments to allow for the uptake of metabolites or the production of required compounds. These dynamics are facilitated by regulation of the rates of individual reactions within the pathways. However, as many intrinsic (e.g., enzyme activity) or extrinsic (e.g., pH or temperature) factors influence these reaction rates, studying cell metabolism remains challenging.

Genome-scale metabolic models (GEMs) can be used to help understand cell metabolism. GEMs represent cell metabolism as a cell-scale network of biochemical reactions, typically distributed over several cellular compartments, that connects the uptake of nutrients to the production of biomass precursors (20, 21). Assuming metabolism to be in steady state allows for the derivation of possible rates (called metabolic fluxes) for each reaction in the network (see File S1 in the supplemental material). A GEM can be used to predict putative nutrients and essential enzymes/reactions in the cell, thereby generating hypotheses about the responses of the cell metabolism to perturbations (22). Therefore, metabolic models have great potential to aid in the development of novel control strategies against pathogens (23, 24). The metabolism of a pathogen is tightly interconnected with that of its host and can be regarded as a single system (25). Moreover, the relationship of a pathogen with its host shapes its metabolism through evolution, leading to enzyme gene loss, which makes the pathogen dependent on its host (26). An integrated host-pathogen metabolic model can yield insights into the system-wide metabolic fluxes that shape an infection. Thus far, only a few models exist that describe the joint metabolism of beneficial or pathogenic microbes and their hosts (27–33).

10.1128/mBio.00454-19.5 FILE S1 Principles of metabolic modeling and explanation of flux coupling analysis. Download TEXT S1, PDF file, 0.2 MB. Copyright © 2019 Rodenburg et al. 2019 Rodenburg et al. This content is distributed under the terms of the Creative Commons Attribution 4.0 International license.

Recently, we published the first GEM for *P. infestans* (34). We used this model to simulate the growth of *P. infestans* on minimal culture medium and predicted the essential genes and corresponding reactions that are required to convert its nutrients into biomass components (34). Here, we integrated the *P. infestans* GEM with a tomato GEM (35). Using these models, we developed a host-pathogen interaction metabolic model that allowed us to identify and study hallmarks of the *P. infestans*-tomato interaction. Exploiting gene expression data of late blight infections on tomato enabled us to dissect the changes in pathogen and host metabolism, providing novel insights into this host-pathogen interaction.

## RESULTS and DISCUSSION

### A metabolic model for the tomato-*P. infestans* pathosystem.

**FIG 1:**
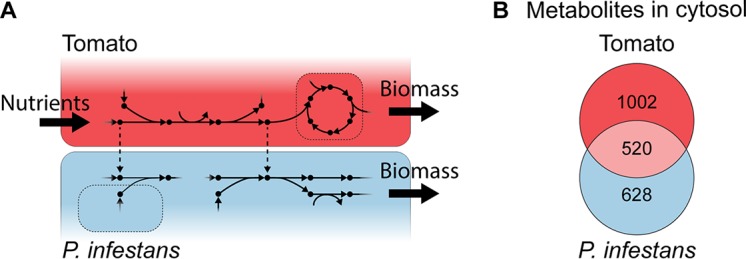
Integrated *P. infestans*-tomato model. (A) Schematic illustration. Dots are metabolites, arrows are reactions, and dotted lines represent the host-pathogen transport reactions. (B) Numbers of unique and shared cytosol metabolites.

To reconstruct an integrated tomato-*P. infestans* metabolic model, we exploited a recently constructed tomato GEM (35). This tomato model comprised 2,143 reactions and 3,410 genes, whereas the previously constructed *P. infestans* GEM comprised 2,394 reactions and 1,301 genes, suggesting that *P. infestans* has less genetic redundancy for metabolic enzymes than does tomato (Table 1). Our original *P. infestans* GEM was based on a framework reconstructed by the annotation of enzyme orthologs in the *P. infestans* genome and linking those to the biochemical reactions in KEGG (34, 36). Based on the literature, we added a minimal growth medium, a set of known biomass precursors, and cellular compartments, and simulated *in vitro* growth (34). The tomato GEM was originally used to simulate photorespiratory fluxes under different conditions and was reconstructed from LycoCyc (35, 37). We manually improved the *P. infestans* and the tomato GEMs (see Materials and Methods). For instance, according to recent insights into the mitochondrial localization of glycolytic enzymes (17), we curated the gene-reaction association of *P. infestans* in this pathway. In the tomato GEM, we curated the thiamine biosynthesis, since *P. infestans* is a thiamine auxotroph (8). The two GEMs were connected by so-called transport reactions, representing the nutrient flux from tomato to *P. infestans*. Although the transporter repertoire of *P. infestans* has been predicted (16), we could not attribute specific substrates to transporters. Most functional annotations lack specificity, and even between oomycete species, transporter substrate specificity can vary (38). Therefore, we chose an unbiased approach, connecting the two GEMs by the addition of a hypothetical unidirectional transport reaction for each of the 520 metabolites that were found to be shared between the cytosol compartments, each one representing the flux of a single metabolite from the tomato cytosol to the *P. infestans* cytosol (Fig. 1). We realized that for a pathogen to maintain homeostasis during infection, fluxes may occur in both directions (i.e., from the host to the pathogen and vice versa). However, the lack of knowledge of this process only allowed us to model host-to-pathogen nutrient fluxes. The 520 added transport reactions predominantly concern metabolites taking part in primary metabolic subsystems (from KEGG). These include amino acid biosynthesis (*n *=* *75), carbon metabolism (*n *=* *61), purine and pyrimidine biosynthesis (*n *=* *51 and 37, respectively), and ABC transporters (*n *=* *42), which indicates that at least 42 metabolites are known ABC transporter substrates. Of the 520 introduced transport reactions, 179 could carry a flux, meaning that the respective metabolites can both be produced by the tomato plant and assimilated by *P. infestans*. The integration of the two GEMs resulted in an integrated metabolic model of *P. infestans*-tomato interaction, comprising a total of 4,695 reactions, 4,303 metabolites, and 4,578 genes (Table 1).

**TABLE 1:** Properties of the genome-scale metabolic models of *P. infestans* and tomato and of the integrated metabolic model

System	Data for model:		
	*P. infestans*^*a*^	*S. lycopersicum* (tomato)^*b*^	*P. infestans* + *S. lycopersicum*^*c*^
Model name	iSR1301	iHY3410	iSR4578
No. of reactions	2,394	2,143	4,695
No. of metabolites	2,685	1,998	4,303
No. of genes	1,301	3,240	4,578
No. of compartments	7	5	12

a Rodenburg et al. (34).

b Yuan et al. (35).

c This study. The numbers in this column are not the exact sum of the two independent models, since several adaptations were made (see Materials and Methods).

### Flux simulations pinpoint nutrients utilized by *P. infestans*.

Given a metabolic model and a metabolic objective for the cell, optimal values for the fluxes through all reactions can be calculated to simulate the production of *P. infestans* biomass precursors through the import of nutrients from the tomato cytosol (39). An objective could be, for instance, to maximize the production of biomass or to produce biomass at minimal enzyme expense. To study the versatility of the model, we composed four scenarios based on different cellular objectives (Table S2). In scenario I, we calculated the minimal set of nutrients *P. infestans* needs to import from tomato to form all its biomass precursors. This was already possible by importing three compounds, thiamine, l-gamma-glutamyl phosphate, and cysteine. While this scenario is unlikely to occur *in planta*, it does illustrate that *P. infestans* has a comprehensive enzyme repertoire, enabling it to form its biomass precursors based on a very small pool of nutrients. In scenario II, we calculated *P. infestans* biomass production using the minimum number of *P. infestans* reactions possible, to simulate growth with minimal enzyme cost for *P. infestans*. In this scenario, 48 nutrients were used, including ammonium, a known growth substrate (40). However, ammonium is an unfavored nitrogen source compared to amino acids (16). Among other imported nutrients in this scenario are the tricarboxylic acid (TCA) cycle intermediates malic acid and succinic acid, for which *P. infestans* has an experimentally verified transporter (38). Organic acids, such as succinic and malic acids, strongly promote *P. infestans* growth *in vitro*, especially in combination with ammonium as a source of nitrogen (41). Notably, 29 of the nutrients in this scenario are direct biomass precursors for *P. infestans*, including 19 amino acids (aspartate being the sole exception) (Fig. S1). In scenario III, we maximized the usage of *P. infestans* reactions to simulate a scenario in which the pathogen maximally exploits its metabolism. This simulation predicted a pool of 29 nutrients, including the inorganic compounds nitrite and hydrogen sulfide. Interestingly, *Phytophthora* spp. retained the complete assimilation pathways for these compounds (16), in contrast to multiple obligate biotrophic oomycetes which lost multiple genes throughout evolution (8). In scenario IV, we combined scenarios I and II, in which we calculated the minimal nutrient uptake combined with minimal usage of *P. infestans* reactions to produce biomass. Since both nutrient import and assimilation into biomass require energy, these processes likely require a trade-off; hence, we anticipate that this is a more realistic scenario. Here, 38 nutrients were used, 16 of which were amino acids.

10.1128/mBio.00454-19.1 FIG S1 Overlap in numbers of imported nutrients in three flux scenarios composed based on different objective functions and the set of *P. infestans* biomass precursors, as follows: Biomass_pinf_, *P. infestans* biomass precursors; Min v_pinf_, scenario II, minimization of *P. infestans* flux; Max v_pinf_, scenario III, maximization of *P. infestans* flux; and Min v_pinf_ ∪ v_transport_, scenario IV, minimize both *P. infestans* and host-pathogen transport flux. The biomass precursors and imported nutrients in each scenario are listed in Table S2. Download FIG S1, TIF file, 0.2 MB. Copyright © 2019 Rodenburg et al. 2019 Rodenburg et al. This content is distributed under the terms of the Creative Commons Attribution 4.0 International license.

The four scenarios described above have opposite objectives (minimize/maximize the fluxes of *P. infestans*) and simulate extreme circumstances not likely found in nature. The true pool of imported nutrients will likely be a combination of the pools predicted in each individual scenario. A comparison of the nutrients imported in all four scenarios revealed sets of nutrients in common between scenarios. There are 14 nutrients found in three out of four scenarios, i.e., II, III, and IV (Fig. S1), including several amino acids, nucleotide precursors, and glycerol 3-phosphate as a lipid precursor. Surprisingly, aspartate is not imported in any of the scenarios, even though it is a biomass precursor of *P. infestans* and serves as a precursor to a variety of other amino acids (42, 43). An oomycete-specific form of aspartate aminotransferase (EC 2.6.1.1) was found that seems to play a key role in pathogenicity in Phytophthora sojae, possibly by balancing nitrogen and carbon metabolism and facilitating the interconversion between amino acids (44). The presence of this enzyme in our model explains why aspartic acid import may not be necessary. Interestingly, thiamine is only imported in scenario III (maximizing the *P. infestans* fluxes), while thiamine pyrophosphate (TPP) is imported in scenarios II and IV (minimizing fluxes). It is assumed that many oomycetes import thiamine to form TPP (8), an essential cofactor in carbohydrate metabolism (45) and in TPP-responsive riboswitches (46). However, related Stramenopiles were found to also grow on thiamine alternatives (47), and possibly, *P. infestans* can import up- or downstream compounds instead. Taken together, these simulations suggest a range of nutrients from tomato that can be effectively assimilated by *P. infestans*. Nutrients in common between the simulated scenarios suggest that these are likely more versatile than others and hence most useful for *P. infestans* to import.

### Network analysis identifies dependencies of *P. infestans* on tomato metabolism.

**FIG 2:**
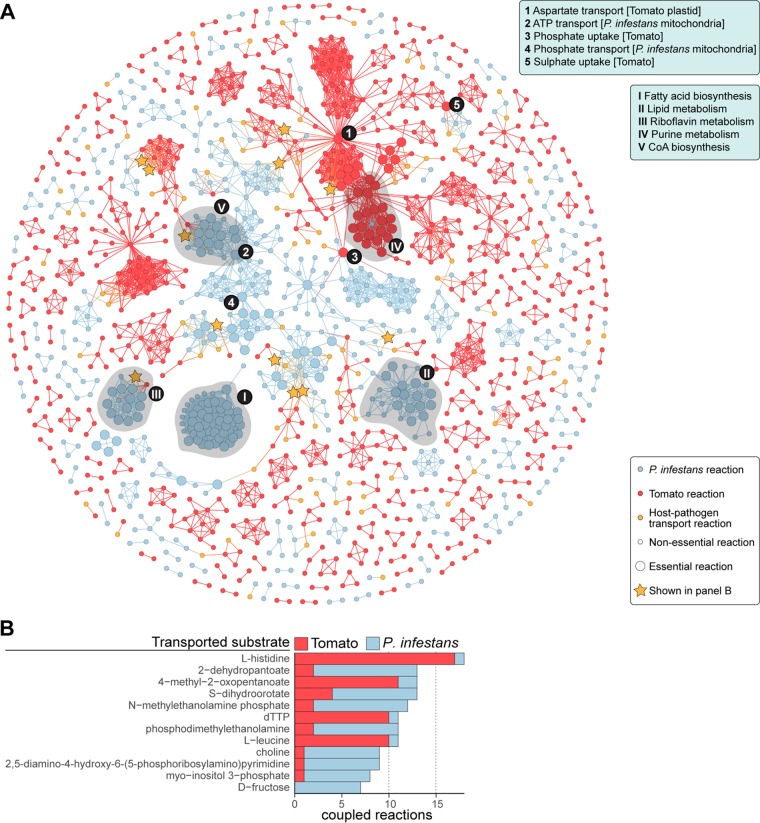
Flux coupling between reactions in the *P. infestans*-tomato model. (A) Graph showing the coupled reactions in the model. Nodes represent reactions in tomato (red) or *P. infestans* (blue) and host-pathogen transport (green), and edges represent coupling between those reactions. Node size reflects essentiality for *P. infestans* biomass production. Stars represent transport reactions listed in panel B. Highly connected nodes (1 to 5) and clusters (I to V) are indicated and listed in the boxes on the right (see also main text). (B) Nutrients associated with the 12 most frequently coupled host-pathogen transport reactions. The bars are stacked and indicate the number of coupled reactions per species.

To identify dependencies of *P. infestans* on tomato, we investigated the topology of the integrated model by looking for essential reactions (i.e., reactions that are indispensable in the model to form all biomass precursors) and (inter)dependencies of reactions (i.e., the flux of a particular reaction relies on the flux of another), also known as coupled reactions. Coupled reactions within a metabolic model can be identified using a method called flux coupling analysis (FCA; File S1) (48). FCA of the model identified 77 coupled *P. infestans*-tomato reaction pairs (Fig. 2) involving 53 unique *P. infestans* reactions and 49 unique tomato reactions (Table S3). One of these *P. infestans*-tomato reaction couplings comprises the biomass reaction of TPP in *P. infestans*, which is coupled to 17 tomato reactions, illustrating that *P. infestans* TPP is dependent on the thiamine biosynthesis pathway in tomatoes. In addition, of the 4,695 reactions in the model, 112 *P. infestans* reactions and 35 tomato reactions were found to be essential for *P. infestans* (Fig. 2A). Some reactions are essential and coupled to many other reactions at the same time (appearing as hubs in Fig. 2A), implying that these play a central role in the model, with potentially large biological implications. For instance, there is a tomato transport reaction of aspartate into the plastid compartment, supplying aspartate as an amino group donor for the synthesis of a thiamine precursor (aminoimidazole ribotide) (49, 50), thus indirectly making this tomato reaction essential for *P. infestans*. Similarly, ATP and phosphate transport between *P. infestans* cytosol and mitochondria is essential and seems to play an important role in the model. Phosphate and sulfate uptake by the tomato plant are part of the defined growth medium, hence coupled to reactions in both species. FCA also revealed several clusters of tightly interconnected coupled essential reactions (Fig. 2A). One of these represents *P. infestans* fatty acid biosynthesis. In the model, this comprises a mostly linear pathway, and consequently, most reactions are coupled (interdependent). Fatty acid biosynthesis in oomycetes is associated with fungicide resistance (51) and energy storage for sporangia (8).

Couplings to the host-pathogen transport reactions in particular can provide information about the importance of transported metabolites; the more *P. infestans* reactions that are coupled to a transport reaction, the more likely that the associated substrate is important for *P. infestans.* We selected the most frequently coupled transport reactions from the model and assessed their couplings to other tomato and *P. infestans* reactions (Fig. 2B). The substrates of these transport reactions were associated with a diverse range of metabolic processes in *P. infestans*, i.e., pantothenate/coenzyme A (CoA) biosynthesis (2-dehydropantoate), *de novo* pyrimidine biosynthesis (*S*-dihydroorotate) (52), riboflavin metabolism [2,5-diamino-4-hydroxy-6-(5-phosphoribosylamino)pyrimidine], and inositol phosphate metabolism (*myo*-inositol 3-phosphate). Notably, three transporter substrates were associated with glycerophospholipid metabolism (phosphodimethylethanolamine N−methylethanolamine phosphate and choline). It is conceivable that *P. infestans* can take up glycerophospholipid precursors to facilitate the formation of membrane lipids (53). Seven *P. infestans* reactions were coupled to the reaction importing fructose, an efficient growth substrate (40). Among the most frequently coupled host-pathogen transport reactions are also those transporting dTTP, histidine, and leucine. Since these are mainly coupled to tomato reactions, the import of these nutrients seems to depend on their biosynthesis pathways in tomato. FCA can identify coupled reactions in metabolic models that are not necessarily directly connected in the network and can thus derive functionally related modules of (pathogen-host) metabolism that may otherwise be overlooked.

### Transcriptome-based submodels provide insight into the dynamics of *P. infestans*-tomato metabolism.

**FIG 3:**
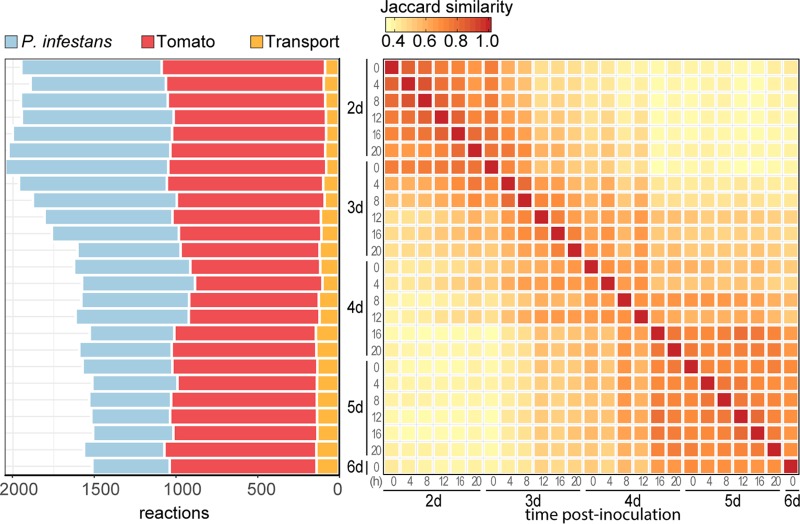
Submodels based on dual-transcriptome data from a time course covering a full infection cycle of *P. infestans* on tomato leaf. Submodels are representative for the sampling time postinoculation shown in days (d) and hours on the *y* axis (middle) and *x* axis (left). Left, stacked bar graph indicating the number of reactions per species and number of host-pathogen transport reactions (not part of either species). Right, Jaccard similarity (intersection divided by union) between the reaction content of the submodels.

To obtain insight into the metabolism at subsequent developmental stages of the *P. infestans*-tomato interaction, we integrated the model with dual-transcriptome data obtained from RNA, isolated from tomato leaves inoculated with *P. infestans* strain 1306 (2 to 6 days postinoculation, sampled every 4 h). The infections showed profuse sporulation at around 4 days postinoculation (dpi). In previous studies, it was found that in infections by this *P. infestans* strain, marker genes for biotrophic growth peak at 2 to 3 dpi, and those for necrotrophic growth peak at 4 to 5 dpi (16), while marker genes for sporulation are increasingly expressed from 3 dpi on and peak at 4 dpi. Expectedly, as the proportion of reads of each sample mapping to the *P. infestans* genome steadily increased over time (from 7% to 84%), the amount of reads mapping to the tomato genome decreased accordingly (from 87% to 8%) (Fig. S2). The transcriptome data were used to generate time point-specific submodels, which are subsets of the full model according to the expression of the genes in the model. Since gene expression and metabolic activity are not directly related, we used the Integrative Network Inference for Tissues (INIT) algorithm (54) that calculates a submodel with maximal agreement to the expression of genes in the model such that biomass can be produced (see Materials and Methods). This resulted in 25 submodels containing 32 to 44% of the reactions of the full model (Fig. 3), indicating that only a subset of reactions is required to form all defined biomass precursors. Over time, the total number of reactions per submodel decreases (Fig. 3), mostly due to a reduction in the number of *P. infestans* reactions. The number of tomato reactions is relatively stable across the submodels, while the number of transport reactions increases slightly over time. These results suggest that as the infection progresses, *P. infestans* relies less on its own metabolism but increasingly imports metabolites from the necrotic tomato lesion. Conceivably, once *P. infestans* switches to its necrotrophic lifestyle, nutrients can be more easily obtained from the decaying leaf tissue (15). To obtain an impression of the metabolic changes in the infection over time, we calculated all pairwise distances of the reaction content of the submodels (Fig. 3). This revealed a gradient of similarity scores, clearly reflecting metabolic changes over time, possibly as a response to changing nutrient availability in the tomato leaf tissue. A relatively large distance can be observed between groups of submodels, suggesting two more profound switches in metabolism at roughly 3 days and 8 h (3d/8h) and 4 days and 12 h (4d/12h), possibly reflecting a transition from the pathogens’ biotrophic growth to necrotrophic growth and sporulation (11, 12, 16, 55).

10.1128/mBio.00454-19.2 FIG S2 Statistics of the RNA sequencing (RNA-seq) read mapping. (A) Per-base quality values for the Illumina reads (all samples combined) derived from the FastQC software. (B) Mapping efficiency per sample for *P. infestans* and tomato. Download FIG S2, TIF file, 1.3 MB. Copyright © 2019 Rodenburg et al. 2019 Rodenburg et al. This content is distributed under the terms of the Creative Commons Attribution 4.0 International license.

**FIG 4:**
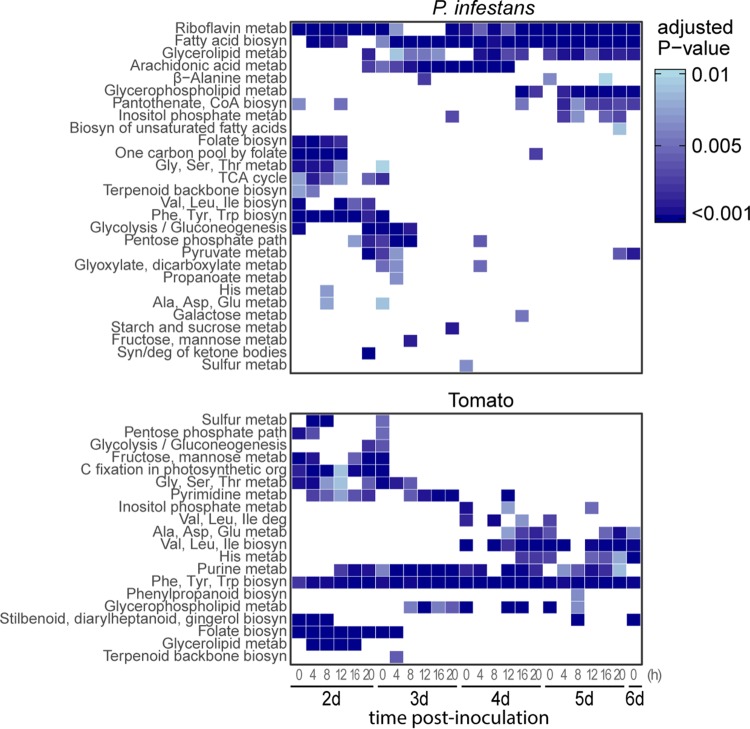
Enriched KEGG pathways in transcriptome-based submodels from a time course covering a full infection cycle of *P. infestans* on tomato. Submodels are representative for the sampling time postinoculation shown on the *x* axis in days (d) and hours. Enrichment is calculated based on the reaction content of each of the submodels compared to the full model. Colors scale to the adjusted *P* value. metab, metabolism; biosyn, biosynthesis; syn, synthesis; deg, degradation.

To obtain more insight into the metabolic processes of each submodel, we performed Fisher’s exact tests on the KEGG pathways in the submodels. A variety of metabolic pathways were overrepresented in submodels compared to the full model (Fig. 4). Consistent with the previously observed gradient of similarity scores (Fig. 3), groups of overrepresented pathways can be distinguished before and after 3d/8h. Notably, in the early submodels prior to the first transition, *P. infestans* reactions are enriched for several primary metabolic pathways, such as amino acid biosynthesis, glycolysis, and the TCA cycle. This could be a response to sugars in the apoplast that are still produced by photosynthesizing tomato leaf cells during the biotrophic phase of infection (56, 57). In contrast, late submodels, and in particular after the second transition (4d/12h), show that in tomato, several amino acid biosynthesis pathways are enriched, suggesting that during early infection, amino acids are mostly synthesized *de novo* by *P. infestans* but are later scavenged from tomato. This corroborates with a transcriptome study of *P. infestans* infecting potatoes, in which a diverse but generally higher expression of amino acid transporter genes at late infection time points was observed (16). In addition, early submodels are enriched in folate-related pathways on both sides, suggesting that folate plays an important role in early infection. This is in line with the finding that *P. infestans* has an unusually large repertoire of putative folate-biopterin transporter genes (18), although a divergent expression pattern was observed for these genes throughout infection (16). Arachidonic acid metabolism is enriched in midinfection submodels, which is a characteristic fatty acid in *P. infestans* known to elicit plant defense and to act as a signaling molecule in plants (58, 59). In summary, our analyses reveal profound changes in metabolic processes during infection of tomato by *P. infestans* and suggest that *P. infestans* reduces its metabolism at the expense of its host.

### Import of specific nutrients becomes increasingly essential to *P. infestans*.

**FIG 5:**
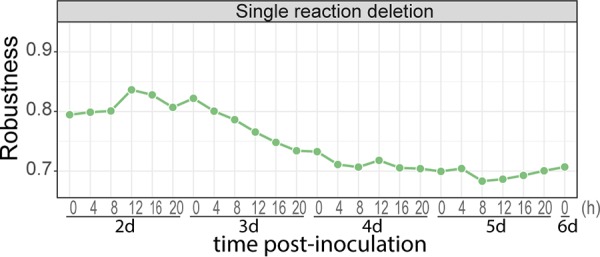
Robustness of transcriptome-based submodels from a time course covering a full infection cycle of *P. infestans* on tomato. The *y* axis shows the robustness as the fraction of single reaction deletions that do not disable the biomass flux for *P. infestans*. Submodels are representative of the sampling time postinoculation shown on the *x* axis in days and hours.

The ability of a model to maintain its functionality (biomass production) under perturbations (e.g., simulated reaction deletions) is often referred to as robustness (60). We can express the robustness of each submodel as the fraction of reaction deletions that do not disturb *P. infestans* biomass production (60). This revealed that early infection submodels have higher robustness to reaction deletions than late infection submodels (Fig. 5). This suggests that as the infection progresses, *P. infestans* largely shuts down accessory/alternative pathways to synthesize its biomass precursors, rendering the remaining reactions essential. The number of essential genes for *P. infestans* remained stable over time, with an average of 84 ± 5 essential genes (Table S1).

10.1128/mBio.00454-19.6 TABLE S1 Reactions in the *P. infestans*-tomato model iSR4578, the associated pathways, formulas, and genes, and their occurrence in the 25 submodels. Reactions that were added during the reconstruction process are listed, as well as essential genes per submodel. Download Table S1, XLSX file, 1.0 MB. Copyright © 2019 Rodenburg et al. 2019 Rodenburg et al. This content is distributed under the terms of the Creative Commons Attribution 4.0 International license.

10.1128/mBio.00454-19.7 TABLE S2 Nutrients imported per scenario (objective function). Download Table S2, XLSX file, 0.01 MB. Copyright © 2019 Rodenburg et al. 2019 Rodenburg et al. This content is distributed under the terms of the Creative Commons Attribution 4.0 International license.

10.1128/mBio.00454-19.8 TABLE S3 Flux coupling analysis results. The cross-species couplings and the flux couplings to transport reactions are shown. Download Table S3, XLSX file, 0.2 MB. Copyright © 2019 Rodenburg et al. 2019 Rodenburg et al. This content is distributed under the terms of the Creative Commons Attribution 4.0 International license.

To evaluate the importance of nutrient transport for *P. infestans* while colonizing tomato, we assessed the essentiality of the host-pathogen transport reactions in each submodel (61). A host-pathogen transport reaction is essential when its deletion disables biomass production and is partially essential when deletion in combination with deletions of other reactions disables biomass production. We found no essential nutrients for *P. infestans* when considering the full model, yet the submodels display various patterns of nutrient essentiality (Fig. S3). Early infection submodels have just a few essential nutrients, while mid- and late-infection submodels, after the first transition point, show various essential amino acid transport reactions. This corroborates our previous observations (Fig. 4) where the first transition point marks the switch from *de novo* synthesis of amino acids in *P. infestans* to an increased uptake from tomato.

10.1128/mBio.00454-19.3 FIG S3 Essentiality score (ESS) for each host-pathogen transport reaction per submodel. The *x* axis represents transcriptome-based submodels, the *y* axis indicates the nutrient that is transported. The transport reactions of nutrients indicated in black are fully essential, and those indicated in various shades of gray are partially essential. Download FIG S3, TIF file, 0.5 MB. Copyright © 2019 Rodenburg et al. 2019 Rodenburg et al. This content is distributed under the terms of the Creative Commons Attribution 4.0 International license.

### Metabolomics can be used to refine transcriptome-based submodels.

**FIG 6:**
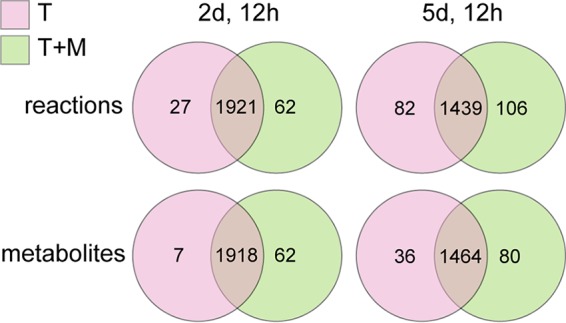
Overlap of reaction and metabolite content of submodels, based on transcriptome data only (T) or transcriptome data and metabolome data (T+M). Data used to generate these submodels were obtained from *P. infestans*-infected tomato leaves at 2d/12h and 5d/12h postinoculation.

To assess to what extent the transcriptome-based submodels are coherent with metabolome data, we utilized untargeted metabolome data of tomato leaves colonized by *P. infestans* at 2 days and 12 h (2d/12h) and 5 days and 12 h (5d/12h) postinoculation. To relate the detected metabolites to metabolic fluxes, we hypothesized that metabolites strongly decreasing in abundance (log_2_ fold change <−2) between these two time points are produced in the submodel of 2d/12h, and metabolites that increase (log_2_ fold change >2) are produced in the submodel of 5d/12h (Fig. S4). Subsequently, we generated two additional submodels using INIT (54) based on the transcriptome data at 2d/12h and 5d/12h postinoculation, while enforcing the presence of the detected metabolites in the respective submodels. The submodels of the transcriptome-only (T) and the metabolome-guided (T+M) submodels differed in 4% (2d/12h) and 11% (5d/12h) of the reactions and in 3% and 7% of the metabolites, respectively (Fig. 6). Overall, the addition of metabolomics data leads to a net increase of reactions and metabolites in particular at the late submodels, suggesting that the T submodels are slightly too conservative; there may be a more active metabolism than suggested by mRNA levels of enzyme-encoding genes. It should be emphasized that both GEMs and metabolomics data only provide a partial description of the cell metabolism at the different infection time points. Discrepancies may arise since transcriptome-based submodels do not take into account any posttranscriptional regulation or posttranslational modification of enzymes, and misannotations and missing/blocked reactions potentially increase uncertainty in submodels. Moreover, since detected metabolites are not necessarily continuously metabolized, steady-state fluxes are not directly linked to measured metabolite abundances (62). Vice versa, metabolic fluxes do not necessarily yield detectable metabolites, for example, when reaction products are immediately consumed in downstream reactions. To predict metabolic fluxes more reliably, higher-resolution metabolome data and sufficient data points over time are prerequisites which could provide sufficient data points in time to calculate metabolite coefficients (63). Nonetheless, our initial results suggest that the integration of high-resolution metabolome data of tomato infection has the potential to refine stage-specific patterns that are embedded in the joint metabolism of the *P. infestans*-tomato interaction.

10.1128/mBio.00454-19.4 FIG S4 Integration of metabolome data in the model. (A) Volcano plot of the metabolome data, highlighting metabolites that show a significant log_2_ fold change of <−2 or >2 between two time points postinoculation, 2d/12h and 5d/12h. The *x* axis represents the log_2_ fold change, and the *y* axis represents the significance with the −log_10_ *P* value. (B) Adding metabolome data leads to an increase or decrease in reactions in various pathways. The analysis was done on two submodels, 2d/12h (left) and 5d/12h (right). Negative bars indicate reactions that were removed in the metabolome-guided (T + M) submodels compared to the transcriptome-only (T) submodels, and positive bars indicate reactions that were added. Download FIG S4, TIF file, 0.8 MB. Copyright © 2019 Rodenburg et al. 2019 Rodenburg et al. This content is distributed under the terms of the Creative Commons Attribution 4.0 International license.

### Conclusions.

Pathogens scavenge metabolites from their hosts to support their growth and proliferation. Here, we took a systems biology approach and modeled the joint metabolism of tomato and *P. infestans* to generate hypotheses about their relationship. Our metabolic model of the *P. infestans-*tomato interaction represents one of the few integrated pathogen-host metabolic models published to date (23). Regarding pathogen and host as one entity can yield hypotheses about the combined metabolism at a single metabolic equilibrium. The modeling allowed us to infer a conceivable pool of nutrients, mainly consisting of amino acids, lipid precursors, and a TPP precursor that is likely exploited by *P. infestans* while infecting the tomato. The model also helped us further characterize host-pathogen dependencies, such as the long-known thiamine dependency of *Phytophthora* spp. (64). Modification of the thiamine biosynthesis pathway in tomato might be an interesting strategy for controlling late blight. Similarly, *P. infestans* lipid metabolism might be an interesting pathway for pathogen intervention, as our model predicts that *P. infestans* takes up lipids as a membrane precursor, coherent with previous experiments (53). Alternatively, fatty acid biosynthesis likely has an important role in synthesizing fuel reserves for spores (8). Our analyses showed that fatty acid biosynthesis is a largely linear pathway with high interdependency of the participating reactions (34).

Clearly, many parameters that can further improve this model are still unknown, for example, the biomass composition of *P. infestans* growing *in planta* and different metabolic processes in different zones of a lesion and during the infection cycle (65). As such, the model would benefit from more extensive and in-depth metabolomics analyses (66). There is also a need to extend our knowledge on the potential role of transporters in this system, as these play a key role in infection and are potential control targets (67, 68). This could be done, for example, by ^13^C-flux spectral analyses to monitor nutrient fluxes and to validate transporter functions in the pathogen (38, 69). Altogether, this model provides insights into *P. infestans*-tomato metabolism and serves as a stepping stone for the design of novel control strategies for this devastating pathogen.

## MATERIALS AND METHODS

### Model reconstruction.

To improve the predictive capabilities of the previously constructed *P. infestans* iSR1301 GEM (34), we performed several literature- and protocol-based curations (70). As a starting point, we used our published genome-scale metabolic model (GEM) of *P. infestans* iSR1301 (34). We removed the sink- and gap-filling reactions that were previously added (34). The sink reactions of the generic metabolites fatty acid, holo-acyl-carrier protein, and apoprotein have no mass or formula but are needed to calculate fatty acid biosynthesis fluxes and were therefore retained. Recently, it was confirmed that some metabolic enzymes of glycolysis and serine biosynthesis in *P. infestans* reside in mitochondria (17). We manually corrected the gene-reaction associations accordingly to assign these enzymes to the appropriate compartment. The KEGG reaction identifiers of the *P. infestans* GEM were matched to MetaNetX identifiers (71), and the associated reaction formula was retrieved. MetaNetX indicates for each reaction whether it is mass balanced or not; all reactions that were not mass balanced were removed from the model. The conversion of arachidonic acid to eicosapentaenoic acid (EPA) was manually added according to the literature, whereafter EPA was added to the biomass precursors (59, 72). Thiamine diphosphate was added to the set of *P. infestans* biomass precursors to represent the thiamine auxotrophy of *P. infestans*. Water and proton metabolites were removed from the model, as they do not fulfill a meaningful function in the *P. infestans* GEM but do have a function in the tomato GEM, where they simulate proton pumps (35). The thermodynamic constraints (directionality) were by default inferred from KEGG maps and manually adjusted according to standard model reconstruction protocols (70), such that ATP/GTP-consuming reactions were unidirectional, except for ATP synthase (EC 3.6.3.14), nucleotide diphosphate kinase (EC 2.7.4.6), and succinate coenzyme A synthase (EC 6.2.1.4).

To identify possible missing enzymes for *P. infestans* in the model, we annotated KEGG enzyme orthologs (KOs) in 19 oomycete proteomes which were downloaded from FungiDB (2 June 2018) (73) (Table S1), as previously described (34). We selected enzymes present in at least two oomycetes and not yet in the model. The encoding protein sequences were aligned to the *P. infestans* T30-4 genome sequence (74), masked for its annotated open reading frames, using tblastn (v2.2.31+). Sequences with an E value of ≤1e^−50^ and query coverage of >90% were submitted to the LocTree3 Web server (75) to predict their subcellular localization, and the associated reactions were added to the model. Additionally, *P. infestans* gene models were manually corrected and reannotated for enzyme orthologs (34). To avoid spurious addition of reactions, any of the added reactions described above were removed if they were eventually found to be unable to carry flux in the model.

The tomato GEM was inferred from supplemental files of the associated publication (35). Since the tomato GEM iHY3510 was built by a different lab using a different reconstruction methodology, we tried to keep adaptations to a minimal level. As was done for the *P. infestans* model identifiers, the reaction identifiers of the tomato model were matched to MetaNetX reaction identifiers for which the associated formula was retrieved. According to knowledge of thiamine biosynthesis in plants (50), the thiamine biosynthesis was manually curated, such that thiamine is formed in the tomato cytosol from a plastidial thiazole and pyrimidine precursor (76). The *P. infestans* and tomato GEMs were connected by unidirectional transport reactions for all metabolites shared between the tomato cytosol and the *P. infestans* cytosol compartments (Table S1).

### Constraint-based modeling and optimization.

Modeling was performed in MATLAB (R2017B) using the RAVEN (v2) (77) and COBRA (v3) toolboxes (78). Flux coupling analysis (FCA) was performed using F2C2 (v0.91) (79). Optimization was performed using Gurobi (v8.0) for flux balance analysis (FBA) and the Integrative Network Inference for Tissues (INIT) (54). GLPK (v2.8) was used for FCA. Essential genes/reactions and essentiality scores of the transport reactions were calculated using ESS (61), which implements Fast-SL (80), constraining the total flux of *P. infestans* biomass production to >5%. The global robustness statistic was calculated according to Peyraud et al. (60), defined as the fraction of reaction deletions that did not render the biomass flux of *P. infestans* <5%.

### RNA sequencing and mass spectrometry.

RNA and metabolites were isolated from tomato leaflets (cv. New Yorker) inoculated with *P. infestans* isolate 1306. For the plant infection assays, a zoospore suspension was prepared as described previously (18) and adjusted to a concentration of 5 × 10^4^ per ml, applied to detached leaves placed on 1.5% water agar. The zoospores were sprayed on the leaves with a hand sprayer until run-off. The leaves were then incubated at 18°C under high humidity in plastic bags containing wet paper towels with a 12-h light/dark cycle. Between 2 and 6 days postinoculation, leaves were harvested every 4 h and flash-frozen until further use. After library construction, single-end 75-nucleotide (nt) sequence reads were obtained using an Illumina NextSeq 500 platform, and quality was assessed using FastQC (v0.11.8) (Fig. S2) (https://www.bioinformatics.babraham.ac.uk/projects/fastqc/). The reads were independently mapped to the *P. infestans* T30-4 (74) and tomato ITAG2.3 (81) genomes using HISAT2 (v2.1.0) (82), and mapping efficiencies were retrieved using SAMtools flagstat (v0.1.19) (83). Transcript abundance was quantified and normalized using cuffnorm (v2.2.1) (84) that implements the DESeq2 normalization procedure (85), which divides the read counts by a factor calculated from the median of geometric means across samples, accounting for differences in sequencing depth and RNA composition.

For the metabolome analyses, the flash-frozen leaves were lyophilized, weighed, and provided to Metabolon, Inc. for further handling. After tissue grinding, proteins were removed by methanol precipitation. After eliminating the solvent, the samples were analyzed by reverse-phase (RP) ultraperformance liquid chromatography (UPLC)-tandem mass spectrometry (MS/MS) using positive-ion mode electrospray ionization (ESI), RP-UPLC-MS/MS with negative-ion mode ESI, and hydrophilic interaction-UPLC-MS/MS with negative-ion mode ESI. The area under the curve method was used to quantify peaks. For each time point, four biological replicates were analyzed. Differential abundance of metabolites was determined using *t* tests, selecting metabolites with a log_2_ fold change of >2 or <−2 with a Benjamini-Hochberg-adjusted *P* value of <0.05 (86). We imputed missing values among replicates using the minimal value across other replicates.

### Submodel generation.

The transcriptome-based submodels were generated using the INIT algorithm (54), which poses a mixed-integer linear optimization problem (MILP) that aims to optimize a global score based on reaction weights in the model. Reaction weights were calculated according to an adapted version of the formula described by Agren et al. (54):

$$w_{\mathrm{ij}}=5log\left(\frac{E_{\mathrm{ij}}}{{\overset{¯}{x}}_{i}\;+\;1}\right),$$

where *w_ij_* is a weight for each reaction *i* and condition *j* in the full model, *E_ij_* is the transcript abundance value summarized per reaction (i.e., the maximum expression value of the genes associated with each reaction), and *x̄*_*i*_ is the mean expression of that reaction across samples. An inflation factor (+1) was added in the denominator to prevent inflated weights for extremely low expression values. According to Agren et al. (54), we maintained the same minimum and maximum cutoffs for *w* of −5 ≤ *w_ij_* ≤ 10. Reactions with an expression lower than the average (thus negative weight) will be less likely to be included in the submodels, and reactions expressed above average (thus a positive weight) will be more likely to be included. Transport reactions were assigned a weight of −0.1, based on the hypothesis that these should not be “free” to include (i.e., weight 0) but should only have a minimal influence on the submodel solution. Reactions without associated genes were given a weight of 0. For the MILP optimization, the flux of all biomass precursors of both tomato and *P. infestans* was constrained to be >1.

### Data availability.

Transcriptome data were deposited in the NCBI Sequence Read Archive under BioProject number PRJNA516028.

10.1128/mBio.00454-19.9 TABLE S4 Gene expression values summarized per reaction and the quantitative metabolome data of the tomato leaf infection. Download Table S4, XLSX file, 0.7 MB. Copyright © 2019 Rodenburg et al. 2019 Rodenburg et al. This content is distributed under the terms of the Creative Commons Attribution 4.0 International license.
