# Supplementary material for: Metabolic Model of the Phytophthora infestans-Tomato Interaction Reveals Metabolic Switches during Host Colonization
Source: mBio. 2019 Jul 9;10(4):e00454-19. doi: 10.1128/mBio.00454-19 (PMC6747730; doi:10.1128/mBio.00454-19)
Supplement: FILE S1 [file mBio.00454-19-s0001.pdf]

Supplementary File S1

Basic principles of metabolic modelling

The reconstruction of genome-scale metabolic models (GEM) yields large networks of interconnected biochemical reactions, typically inferred from homology with known metabolic enzymes in biochemical databases such as KEGG (1), MetaCyc (2) or BRENDA (3). This may be complemented with general or species-specific biological knowledge of metabolism. Reactions in a GEM have a certain stoichiometry, i.e. the balance of metabolites that are consumed and produced in the reaction. For instance, in the reaction of acetyl-CoA C-acetyltransferase (EC 2.3.1.9),

1 CoA + 1 acetoacetyl-CoA ⇄ 2 acetyl-CoA

one CoA and one acetoacetyl-CoA are consumed and converted into two acetyl-CoA molecules or vice versa. A downstream reaction may again consume acetyl-CoA and convert it into another metabolite. In a GEM, the stoichiometry of each reaction is stored in a stoichiometric matrix *S*, which is sparse and has dimensions $m \times n$, where m is the number of metabolites and *n* is the number of reactions (4). The value of each entry S_{ij} is the stoichiometry of metabolite i in reaction j. When i is consumed in j, S_{ij} is given a negative value, when it is produced a positive value.

Each reaction j in a GEM has a flux value v_j, which is defined as the rate of that reaction in steady-state. Steady-state is (often) the central assumption in a GEM, based on the idea that in the long run, metabolites in a cell cannot accumulate or deplete, since cells must maintain homeostasis to proliferate (5). In a GEM, the steady-state assumption implies that the net production and consumption of all metabolites in the model is zero. In other words, the net uptake of nutrient mass into the system must also be produced in the form of biomass.

In mathematical terms, the steady-state of a GEM is described as:

$$S \cdot v = 0$$

where v is the vector of all flux values. Generally, solving this system of equations yields a large number of solutions; that is, it does not result in a single, unique set of fluxes v. Therefore, it is often additionally assumed that the cell attempts to optimize a certain objective, such as maximize growth, minimize energy consumption etc. (6). Given a GEM, one can then find the set of fluxes that optimize the selected objective. This can be posed as a linear optimization program:

$$\begin{aligned} \text{Maximize} \quad & v_{biomass} \\ \text{Subject to:} \quad & S \cdot v = 0 \\ & lb_j \leq v_j \leq ub_j \end{aligned}$$

Here $v_{biomass}$ specifies the flux of one or more biomass reactions that consume the precursor metabolites for all main cell components. The optimal flux values in the solution are limited by the steady-state constraint explained earlier, and thermodynamic constraints that specify for each flux upper- and lower bounds, lb and ub respectively. lb_j can be less than zero, indicating a reverse flux is allowed, representing bidirectional or reverse reactions. Thermodynamic constraints are usually applied for certain reactions by specifying that a reaction may only occur in one direction, thus setting lb or ub to zero.

Linear optimization can be performed by specialized software, such as Gurobi, CPLEX, Mosek or GLPK, that can optimize a GEM consisting of thousands of reactions in a matter of seconds.

Flux coupling

Flux coupling analysis (FCA) is a method that finds coupled fluxes in a GEM. Three basic types of flux coupling were developed (6, 7). FCA finds for each pair of unblocked fluxes in the model v_a and v_b whether they are:

I) Directionally coupled: $v_a \neq 0 \rightarrow v_b \neq 0$. In general terms, any nonzero flux for v_a implies nonzero flux for v_b but not necessarily the reverse. This coupling can hold independently for the other direction: $v_b \neq 0 \rightarrow v_a \neq 0$.

II) Partially coupled: $v_a \neq 0 \leftrightarrow v_b \neq 0$, but not at a constant ratio $v_a/v_b \neq c$.

III) Fully coupled: $v_a \neq 0 \leftrightarrow v_b \neq 0$ and in a constant ratio $v_a/v_b = c$.

In any case, a coupling between two fluxes in a steady-state GEM indicates that flux of one reaction at least partially relies on another, indicating a functional relationship between the associated reactions.

1. Kanehisa M, Sato Y, Kawashima M, Furumichi M, Tanabe M. 2015. KEGG as a reference resource for gene and protein annotation. Nucleic Acids Res 44:gkv1070.

2. Caspi R, Altman T, Billington R, Dreher K, Foerster H, Fulcher CA, Holland TA, Keseler IM, Kothari A, Kubo A, Krummenacker M, Latendresse M, Mueller LA, Ong Q, Paley S, Subhraveti P, Weaver DS, Weerasinghe D, Zhang P, Karp PD. 2014. The MetaCyc database of metabolic pathways and enzymes and the BioCyc collection of Pathway/Genome Databases. Nucleic Acids Res 42:D459-71.

3. Placzek S, Schomburg I, Chang A, Jeske L, Ulbrich M, Tillack J, Schomburg D. 2017. BRENDA in 2017: New perspectives and new tools in BRENDA. Nucleic Acids Res 45:D380–D388.

4. Lewis NE, Nagarajan H, Palsson BO. 2012. Constraining the metabolic genotype-phenotype relationship using a phylogeny of in silico methods. Nat Rev Microbiol 10:291–305.

5. Nielsen J. 2017. Systems biology of metabolism. Annu Rev Biochem 86:245–275.

6. Burgard AP, Nikolaev E V., Schilling CH, Maranas CD. 2004. Flux coupling analysis of genome-scale metabolic network reconstructions. Genome Res 14:301–312.

7. Larhlimi A, David L, Selbig J, Bockmayr A. 2012. F2C2: a fast tool for the computation of flux coupling in genome-scale metabolic networks. BMC Bioinformatics 13:57.
