# Supplementary figures and images for: Metabolic Model of the Phytophthora infestans-Tomato Interaction Reveals Metabolic Switches during Host Colonization
Source: mBio. 2019 Jul 9;10(4):e00454-19. doi: 10.1128/mBio.00454-19 (PMC6747730; doi:10.1128/mBio.00454-19)

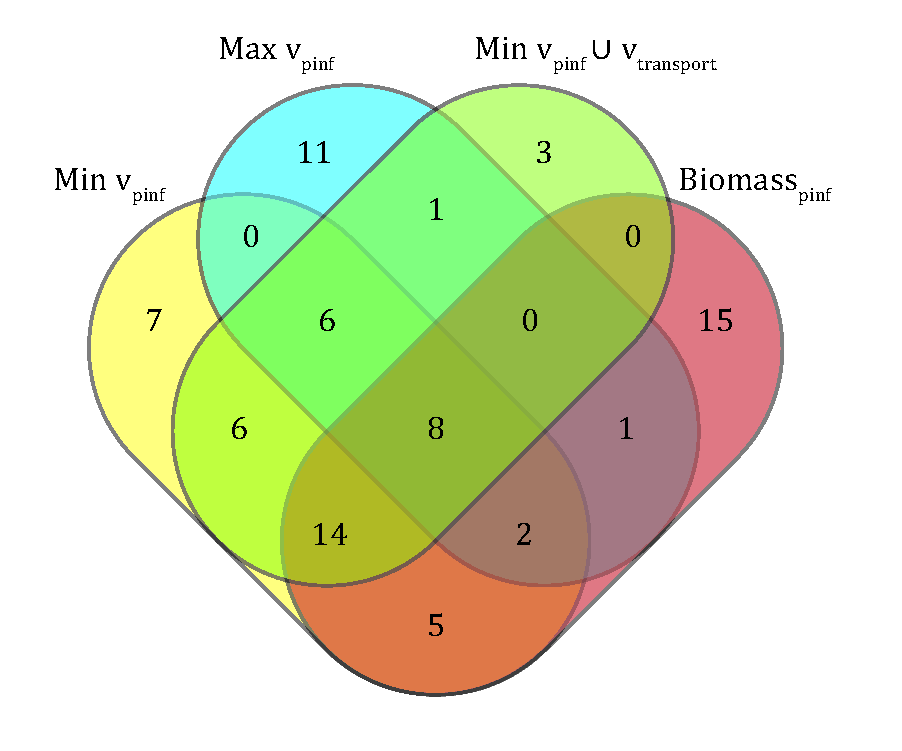

Supplement: FIG S1 [file mBio.00454-19-sf001.tif]

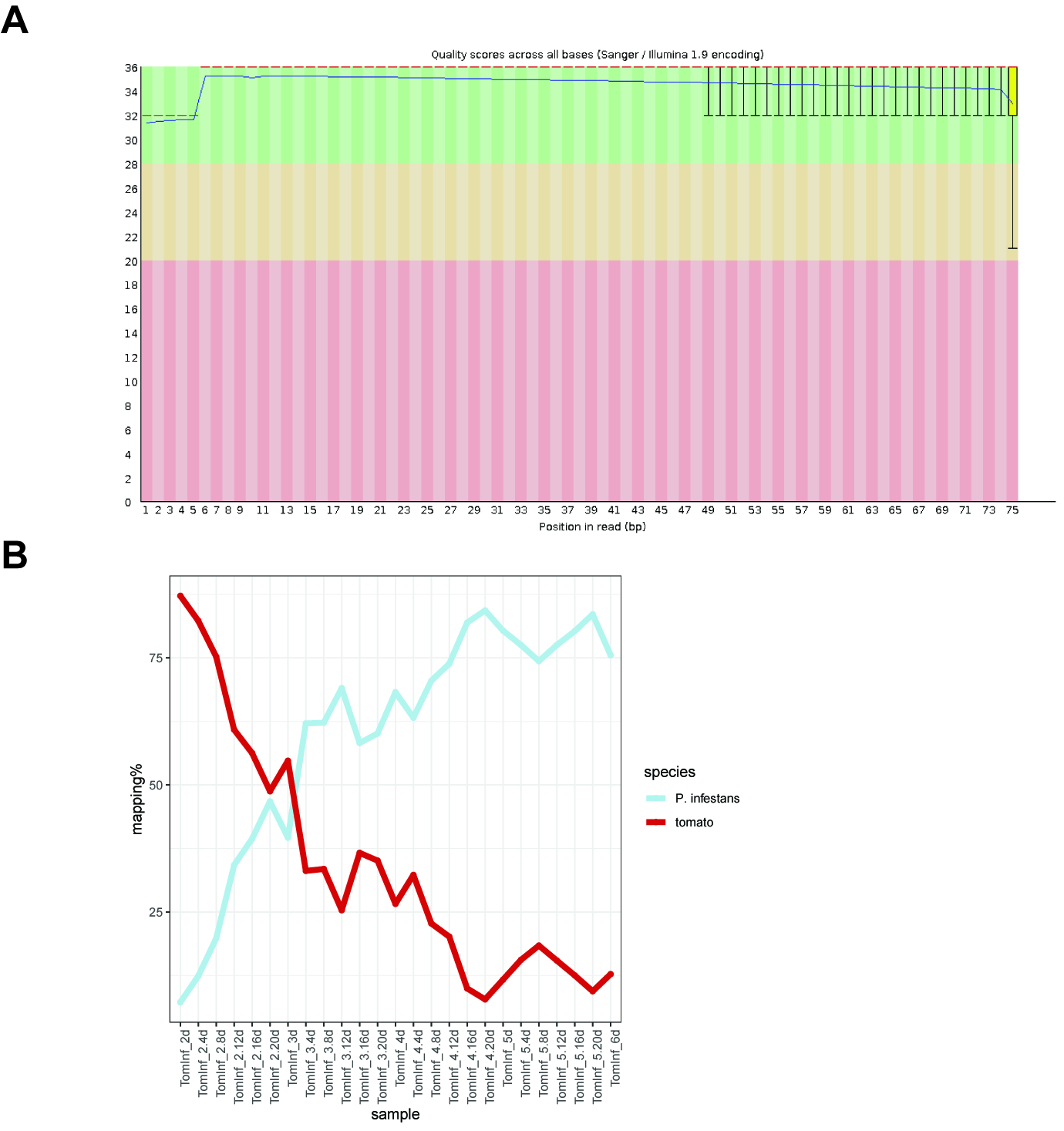

Supplement: FIG S2 [file mBio.00454-19-sf002.tif]

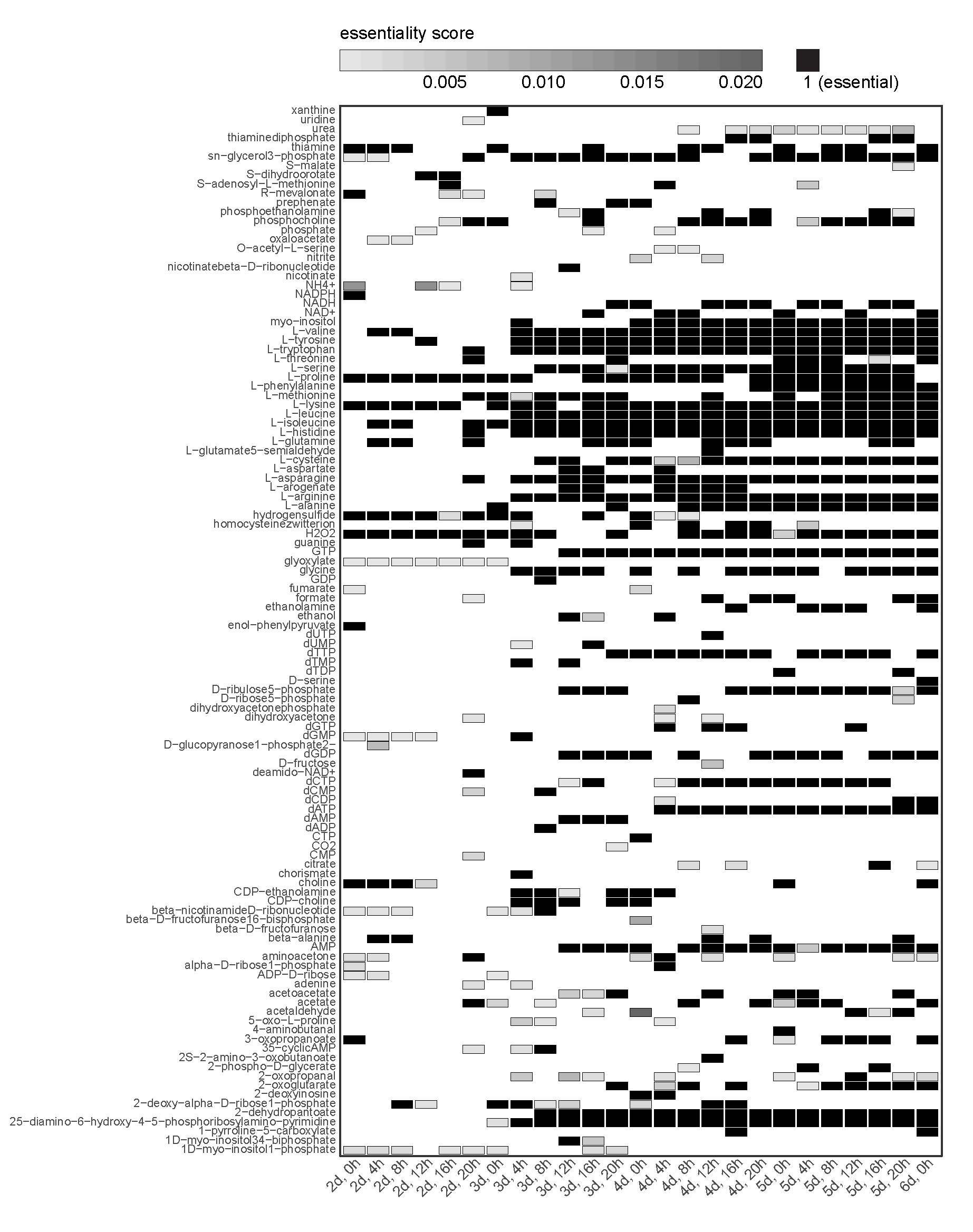

Supplement: FIG S3 [file mBio.00454-19-sf003.tif]

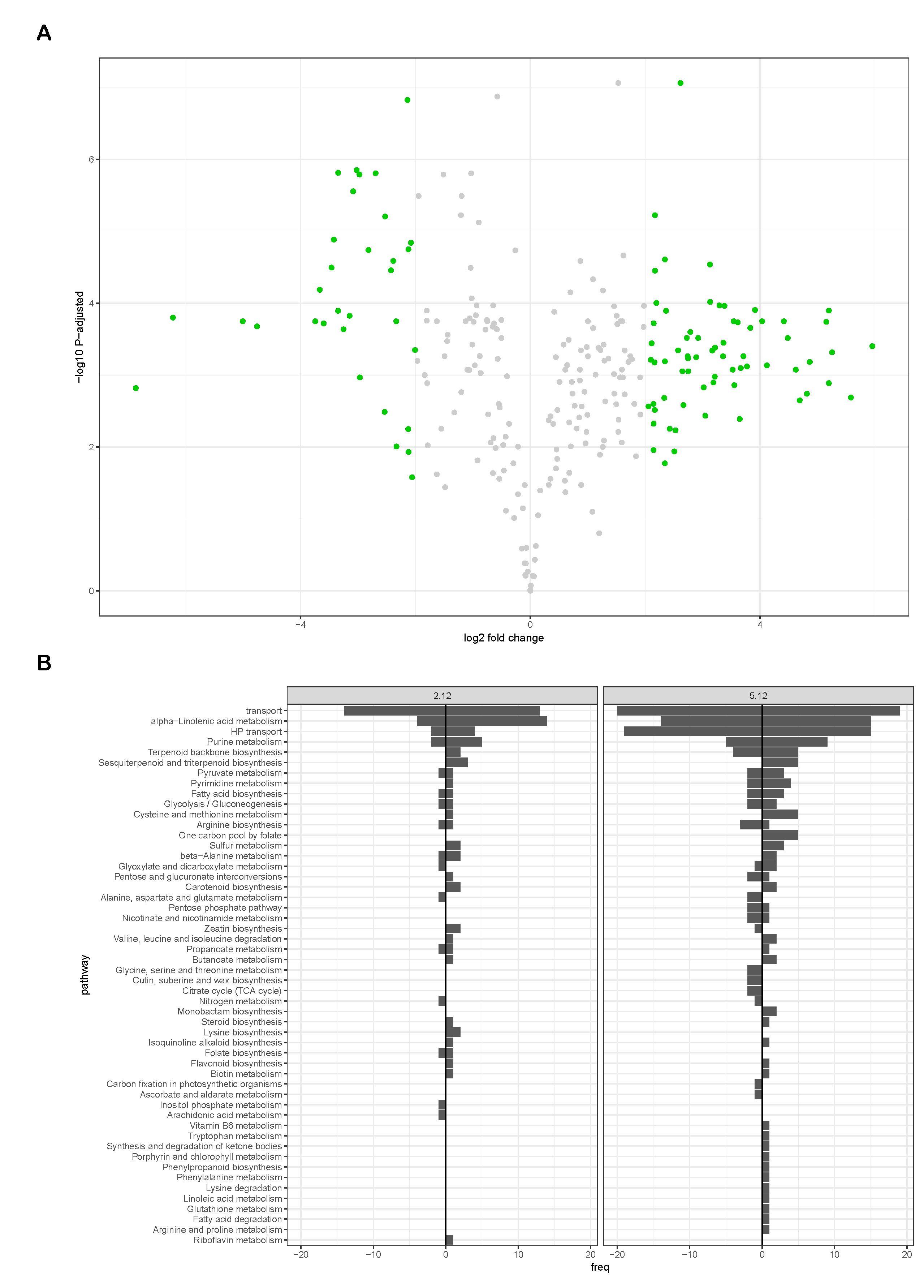

Supplement: FIG S4 [file mBio.00454-19-sf004.tif]
